# Defining the commercial determinants of health: a systematic review

**DOI:** 10.1186/s12889-020-09126-1

**Published:** 2020-06-29

**Authors:** Cassandra de Lacy-Vawdon, Charles Livingstone

**Affiliations:** School of Public Health and Preventive Medicine, Monash University, Melbourne, Australia

**Keywords:** Commercial determinants of health, Global health, Non-communicable diseases

## Abstract

**Background:**

Despite increasing attention to the social determinants of health in recent decades, globally there is an unprecedented burden from non-communicable diseases (NCDs). Recently, the corporate and commercial conditions associated with these, commercial determinants of health (CDoH), have also begun to receive attention. This research aims to articulate the CDoH as described in the literature, summarize substantive findings, and assess strengths and limitations of current literature.

**Methods:**

Systematic review of formal (Medline, EMBASE, Scopus, Global Health) and grey literature (database, Google Advanced, targeted website, citation searching). Searching identified 125 texts for full-text review, with 33 included for final review. Data extracted were analyzed thematically.

**Results:**

The dynamics constituting CDoH include broad facilitators such as globalization of trade, corporate structures, and regulatory systems, articulation of social and economic power, neoliberal and capitalist ideologies; additional elements include corporate activities such as marketing, corporate political activities, corporate social responsibility, extensive supply chains, harmful products and production, and issues of accessibility. These contribute significantly to worsened global health outcomes.

**Conclusions:**

Literature describing effects of macro conditions and corporate activities on health could usefully utilize CDoH terminology. Facilitation via revised, consistent and operational definition of CDoH would assist. Social, political, commercial and economic structures and relations of CDoH are under-theorized. Systematic approaches to identifying, describing, and disrupting these are required to improve global health.

## Background

Recently, research addressing social determinants of health (SDoH), following formative work by Marmot and colleagues [1], has focused on identifying, describing, and beginning to address underlying social causes of population ill-health. However, SDoH approaches have yet to achieve far-reaching population health improvements. Globally, an unprecedented burden from non-communicable diseases (NCDs) has developed [2]. NCDs are amongst the most pressing contemporary challenges to human health, affecting both high-income and low- and middle-income countries alike, contributing to the double-burden of disease [3].

NCDs are often termed ‘lifestyle diseases’ given their origins in behaviors including diet, physical inactivity, alcohol use, and tobacco use [4, 5]. However, these behaviors are increasingly recognized as socially constructed choices heavily influenced by commercial interests [6, 7]. Some call NCDs ‘industrial epidemics’ [8–12] or ‘profit-’ or ‘corporate-driven diseases’ [12–17] given the prominent involvement of commercial interests, entities and products. Meanwhile, others describe commercial conditions that influence health as ‘corporate’ or ‘commercial determinants of health’ (CDoH). Some researchers have called for CDoH to be afforded the same priority for disease prevention and research priority as SDoH [5, 9, 18], although some may view CDoH as a subset of SDoH.

Despite increasing references to CDoH in the literature, to date, no systematic synthesis of the CDoH literature base has been produced. This review seeks to address this and distil the current CDoH evidence base. This systematic review aims to:

1. Articulate how CDoH, and the prevention or minimization of harm associated with these, have been described in the literature;
2. Summarize substantive findings from identified research; and,
3. Assess the strengths and limitations of identified literature.

The working CDoH definition guiding initial stages of this review was that proposed by Kickbusch, Allen and Franz: “strategies and approaches used by the private sector to promote products and choices that are detrimental to health” [19].

## Methods

This review was conducted in accordance with the Preferred Reporting Items for Systematic Reviews and Meta-Analyses (PRISMA) statement [20]. No protocol has been registered or published elsewhere. Peer-reviewed literature and/or grey literature providing a definition and/or description of the drivers or underlying causes, channels or mechanisms, and/or outcomes associated with CDoH, were included.

Original studies, reviews, commentaries, editorials, discussion papers, books and book chapters, reports, web articles and resources from government and non-government organizations, and conceptual works were included, where they met inclusion criteria. Other works such as newspaper and online news articles, presentations or speeches, and social media posts were excluded. Data could be qualitative, quantitative, or mixed-method.

Papers naming CDoH directly, and/or describing similar concepts such as ‘corporate determinants’ or corporations and associated practices as social determinants of disease, ill-health, or NCDs were included. Whilst there is an expanse of literature that could be categorized as CDoH, including literature that does not name the CDoH or associated terms explicitly, this review sought a narrower framing to focus in literature self-identified as pertaining to CDoH.

### Search strategy

The search strategy was fivefold and developed with the assistance of two specialist librarians. First, Medline (Ovid), EMBASE (Ovid), Scopus, and Global Health databases were searched. Second, grey literature databases, including Community Guides (CDC), National Institute for Health and Care Excellence, Centre for Reviews Dissemination (University of York), and Health Evidence (Canada) were searched. Third, further grey literature searching was conducted using Google Advanced Searches consistent with systematic grey literature searching approaches described elsewhere [21, 22]. Fourth, targeted grey literature searching of key organizations’ websites, consistent with previous approaches, was conducted [21, 22]. Finally, backwards and forwards citation searches were completed. See ‘Additional file 1 - Search strategies’ for complete search strategies.

An iterative approach and preliminary search testing indicated appropriate search terms. A keyword search was adopted to capture the relevant CDoH literature ((commercial OR corporate).mp AND (determinant*.mp) AND (health OR disease*).mp). Databases were searched 15 May 2018, and all results were exported, duplicates removed, and screened using EndNote X8 software. Grey literature searches were conducted in June 2018, with results screened online, and relevant full-texts imported to EndNote. Consistent with previous studies the first 100 results for web searches were screened [22]. Citation searches were initially conducted 17 August 2018 and updated 29 March 2019.

### Literature selection

Titles and abstracts were screened for all search results. Where abstracts were not available, executive summaries and/or tables of contents were used. Literature was excluded where it: was not published in English; included data and/or findings relating to non-humans; presented modelling, clinical and/or laboratory findings without examining underlying determinants; or, presented descriptive findings from population-level disease or risk behavior surveillance and/or public health or health promotion interventions without examining underlying determinants. We included literature that provided a definition and/or description of CDoH, either naming these directly, or describing these indirectly as underlying determinants of health and/or disease as described previously.

Following screening, full texts were retrieved, with literature excluded where full texts were unavailable. Full texts were independently reviewed by two reviewers, and tabulated by one reviewer. Discrepancies were resolved via consultation between reviewers. Literature failing to meet inclusion criteria was excluded. Data extracted included author(s), date, title, publisher/source, type of publication, type of evidence provided, underpinning theories and/or frameworks used to frame analysis, and CDoH terms used (see Table 1).

**Table 1:** Literature characteristics. Characteristics of the included literature

Author (Year)	Title	Publisher (Document type)	Type of evidence	Underpinning theory/ framework used to frame analysis	Commercial Determinants of Health term(s) used
Buse & Hawkes (2015) [13]	Health in the Sustainable Development Goals: ready for a paradigm shift?	Globalization and Health (Journal article)	Narrative and descriptive review of evidence base for the health-related targets in the (then) proposed Sustainable Development Goals in relation to disease burden and feasibility of interventions to achieve targets.	None	Commercial determinants of ill-health; and ‘Profit-driven-diseases’ and their commercial determinants
Buse, Tanaka & Hawkes (2017) [16]	Healthy people and healthy profits? Elaborating a conceptual framework for governing the commercial determinants of non-communicable diseases and identifying options for reducing risk exposure	Globalization and Health (Journal article)	Narrative and descriptive analysis of conceptual framework and related health governance literature.	Uses an existing conceptual framework designed to classify the involvement of the commercial sector in global governance for health. The framework presents three models of interaction between public and private sectors: self-regulation by industry; regulation through partnership; and regulation of the private sector by the public sector.	Commercial determinants of NCDs; commercial determinants of health; and commercial determinants of ill-health
Collins, Mikkelsen, & Axelrod (2019) [23]	Interact, engage or partner? Working with the private sector for the prevention and control of noncommunicable diseases	Cardiovascular Diagnosis and Therapy (Journal article)	Narrative and descriptive paper describing the role of the private sector in noncommunicable disease prevention and control	None	NCD risk factors and their underlying social and commercial determinants
Franz & Kickbusch (2018) [24]	The Capital-NCD-Nexus: The commercial determinants of health and global capital flows	Eurohealth (Journal article)	Narrative and descriptive article discussing the role of global capital flows for health and noncommunicable diseases	None	Commercial determinants of health
Freudenberg & Galea (2008) [6]	The impact of corporate practices on health: Implications for health policy	Journal of Public Health Policy (Journal article)	Narrative and descriptive case studies (*n* = 3) of trans fats, sports utility vehicles, and a painkiller to examine the role of corporate policies and practices in the production of health and disease, and suggest policy implications.	None	Corporations as a social determinant of health
Freudenberg & Galea (2007) [25]	Corporate Practices (In Macrosocial Determinants of Population Health)	Springer (Book Chapter)	Narrative and descriptive literature review of corporate practices that harm health with proposed conceptual model focusing on six industries.	Presents an original conceptual model of the influences of corporate practices on health.	Corporations as a social determinant of health
Hastings (2015) [9]	Public health and the value of disobedience	Public Health (Journal article)	Narrative and descriptive application of concepts from historic philosophical writings to modern day public health challenges, including corporate marketing.	Uses Etienne de la Boétie’s work on ‘Voluntary Servitude’ to explore power and public health.	Commercial determinants of ill health
Hastings (2012) [5]	Why corporate power is a public health priority	BMJ (Journal article)	Narrative and descriptive discussion of corporate power, and especially corporate marketing, as a public health priority.	None	Commercial determinants of ill-health
International Federation of Medical Students’ Associations, Team of Officials (2017) [26]	IFMSA Policy Non-Communicable Diseases	International Federation of Medical Students’ Associations (Policy statement)	Policy statement incorporating discussion of literature as rationale (evidence) for position.	None	Commercial determinants of health
Ireland et al. (2019) [27]	Commercial determinants of health and sport sponsorship	Bulletin of the World Health Organization (Journal article)	Narrative and descriptive discussion of sport sponsorship as a commercial determinant of health	None	Commercial determinants of health
Kadandale, Marten, & Smith (2019) [28]	The palm oil industry and noncommunicable diseases	Bulletin of the World Health Organization (Journal article)	Narrative and descriptive paper using the Kickbusch et al. (2016) CDoH framework to frame analysis of the palm oil industry	Uses Kickbusch et al. (2016) commercial determinants of health framework to frame analysis	Commercial determinants of health
Kickbusch (2015) [14]	Addressing the commercial determinants is critical to emerging economies	Ciencia & Saude Coletiva (Journal article)	Narrative and descriptive brief article describing need for emerging economies to take the lead in addressing the commercial determinants of health due to the unequal effect on these societies.	None	Commercial determinants of health
Kickbusch (2013) [29]	A Game Change in Global Health: The Best Is Yet to Come	Public Health Reviews (Journal article)	Narrative and descriptive article discussing the need for a better-equipped (health) governance system to improve health, address commercial determinants, and reduce inequalities.	None	Commercial determinants of NCDs; and commercial determinants of health
Kickbusch (2012) [18]	Addressing the interface of the political and commercial determinants of health	Health Promotion International (Journal editorial)	Narrative and descriptive article describing the need to address the political and commercial determinants of health in order to continue to move the health agenda forward.	None	Commercial determinants of health
Kickbusch, Allen & Franz (2016) [19]	The commercial determinants of health	The Lancet Global Health (Journal article)	Narrative and descriptive article to introduce a new definition of the commercial determinants of health and present an associated framework.	Presents and describes a framework depicting the dynamics that constitute the commercial determinants of health.	Commercial determinants of health
Kickbusch & Szabo (2014) [30]	A new governance space for health	Global Health Action (Journal article)	Narrative and descriptive article describing need for global public goods for health and a rules-based and reliably financed global public health domain to promote global health.	None	Commercial determinants of global health; and commercial determinants of health
Knai et al. (2018) [31]	Systems Thinking as a Framework for Analyzing Commercial Determinants of Health	The Millbank Quarterly (Journal article)	Narrative and descriptive drawing on a systems thinking framework to frame discussion	Uses Donella Meadows’s systems thinking framework	Commercial determinants of health; commercial determinants of NCDs
Kosinska & Ostlin (2016) [32]	Building systematic approaches to intersectoral action in the WHO European Region	Public Health Panorama (Magazine editorial)	Narrative and descriptive overview of the magazine issue and the commercial determinants of health, with reference to the Sustainable Development Goals and the Health 2020 agenda.	None	Commercial determinants of health
Madureira Lima & Galea (2018) [33]	Corporate practices and health: a framework and mechanisms	Globalization and Health (Journal article)	Narrative and descriptive article to introduce a framework for mapping corporate activity.	Uses Steven Lukes’s three-dimensional view of power to study the practices deployed by commercial interests to foster consumption. Presents a framework to study corporations and commercial interests as a distal, structural, societal factor that causes disease and injury.	Deaths worldwide … attributable to behavioral risk factors that, at their core, have the consumption of unhealthful products and exposures produced by profit driven commercial entities; and commercial interests as distal, structural, societal factors that cause disease and injury
McKee & Stuckler (2018) [34]	Revisiting the Corporate and Commercial Determinants of Health	American Journal of Public Health (Journal article)	Narrative and descriptive article outlining the emergence of the commercial determinants of health, how corporations influence health, and how public health professionals can respond to this power.	None	Corporate and commercial determinants of health
Millar (2013) [15]	The corporate determinants of health: How big business affects our health, and the need for government action!	Canadian Journal of Public Health (Journal article)	Narrative and descriptive commentary describing the effect corporations have on health in Canada and the government action needed to protect consumers and reduce harm.	None	Corporate determinants of health
Public Health Association of Australia (2018) [35]	What are the determinants of health?	Public Health Association of Australia (Web article)	Narrative and descriptive document describing the determinants of health, including the social, ecological, political, commercial, and cultural determinants with reference to relevant literature.	None	Commercial determinants of health
Smith, Buse & Gordon (2016) [36]	Civil society: the catalyst for ensuring health in the age of sustainable development	Globalization and Health (Journal article)	Narrative and descriptive article using illustrative examples to discuss how civil society can contribute to global health.	None	Commercial determinants of health
Smith, Dorfman, Freudenberg, Hawkins, Hilton, Razum & Weishaar (2016) [37]	Tobacco, Alcohol, and processed Food industries – Why Do public Health practitioners View Them So Differently?	Frontiers in Public Health (Journal article)	Narrative and descriptive opinion piece on how public health should engage with commercial interests in tackling the NCD epidemic.	None	Social determinants of NCDs
Sula-Raxhimi, Butzbach, & Brousselle, (2019) [38]	Planetary health: countering commercial and corporate power	The Lancet Planetary Health (Journal article)	Narrative and descriptive and presents a framework for countering the effects of corporate power and commercial determinants of health	Presents a framework for countering the effects of corporate power and commercial determinants of health, inspired by ecological determinants of health and commercial determinants of health frameworks	Commercial determinants of health
Thorn (2018) [39]	Addressing power and politics through action on the commercial determinants of health	Health Promotion Journal of Australia (Journal article)	Narrative and descriptive opinion piece on power and politics as relevant to the commercial determinants of health	None	Commercial determinants of health
Thurley (2017) [40]	Explaining the links between Commercial Determinants of Health and Chronic Diseases	European Public Health Alliance (Web editorial)	Narrative and descriptive editorial introducing and contextualizing the commercial determinants of health and promoting the European Public Health Alliance 2017 Annual Conference.	None	Commercial determinants of health
United Nations Department of Economic and Social Affairs, and United Nations Industrial Development Organization (2016) [41]	Report of the expert meeting in preparation for HLPF 2017 on readying institutions and policies for integrated approaches to implementation of the 2030 Agenda	United Nations Department of Economic and Social Affairs, and United Nations Industrial Development Organization (Meeting report)	Descriptive report highlighting the issues raised during the meeting attended by representatives from UN Member States, international organizations, academia and major groups and other stakeholders, with a focus on the Sustainable Development Goals.	None	Corporate determinants of health
West & Marteau (2013) [42]	Commentary on Casswell (2013): The commercial determinants of health	Addiction (Journal article)	Narrative and descriptive commentary in response to Casswell ^58^ describing the commercial determinants of health in relation to the alcohol industry.	None	Commercial determinants of health
Wiist (2006) [43]	Public health and the anticorporate movement: Rationale and recommendations	Government, Politics, and Law (Journal article)	Narrative and descriptive article discussing the possible links between the anti-corporate movement and public health in order to improve health.	None	The corporate entity as a social structural determinant of disease
World Health Organization (2017) [44]	2. Convening to overcome commercial determinants of health (In Report of the Regional Director: The work of WHO in the Western Pacific Region 1 July 2016–30 June 2017)	World Health Organization (Report section)	Narrative and descriptive chapter describing the effect of the commercial determinants of health on diet and the need to address these to improve health outcomes.	None	Commercial determinants of health
World Health Organization Regional Office for Europe (2016) [45]	Good governance for the health and well-being of all children and adolescents	World Health Organization Regional Office for Europe (Conference paper)	Narrative and descriptive thematic paper describing the need for governance to promote health and wellbeing in children and adolescents.	None	Commercial determinants of health

Source: Texts included for systematic review

### Quality assessment

As CDoH represent an emerging research field, it was anticipated that literature would be primarily descriptive and conceptual rather than rich in original data and analyses. Applying risk of bias assessment tools (e.g., Cochrane Handbook for Systematic Reviews [46] or the Consolidated Criteria for Reporting Qualitative Research [47]) was therefore inappropriate. Instead, type and source of publication, type of evidence used, and any underpinning frameworks or theories were appraised. Broad comments on evidence quality are included.

### Synthesis of results

Meta-analysis was not appropriate. Thematic analysis using an inductive approach to the generation of themes and sub-themes, framed by the research aims, was adopted. This process involved stages of coding and summarizing thematic material and presenting these to form a novel synthesis of the current literature.

## Results

### Literature selection

**Fig. 1:**
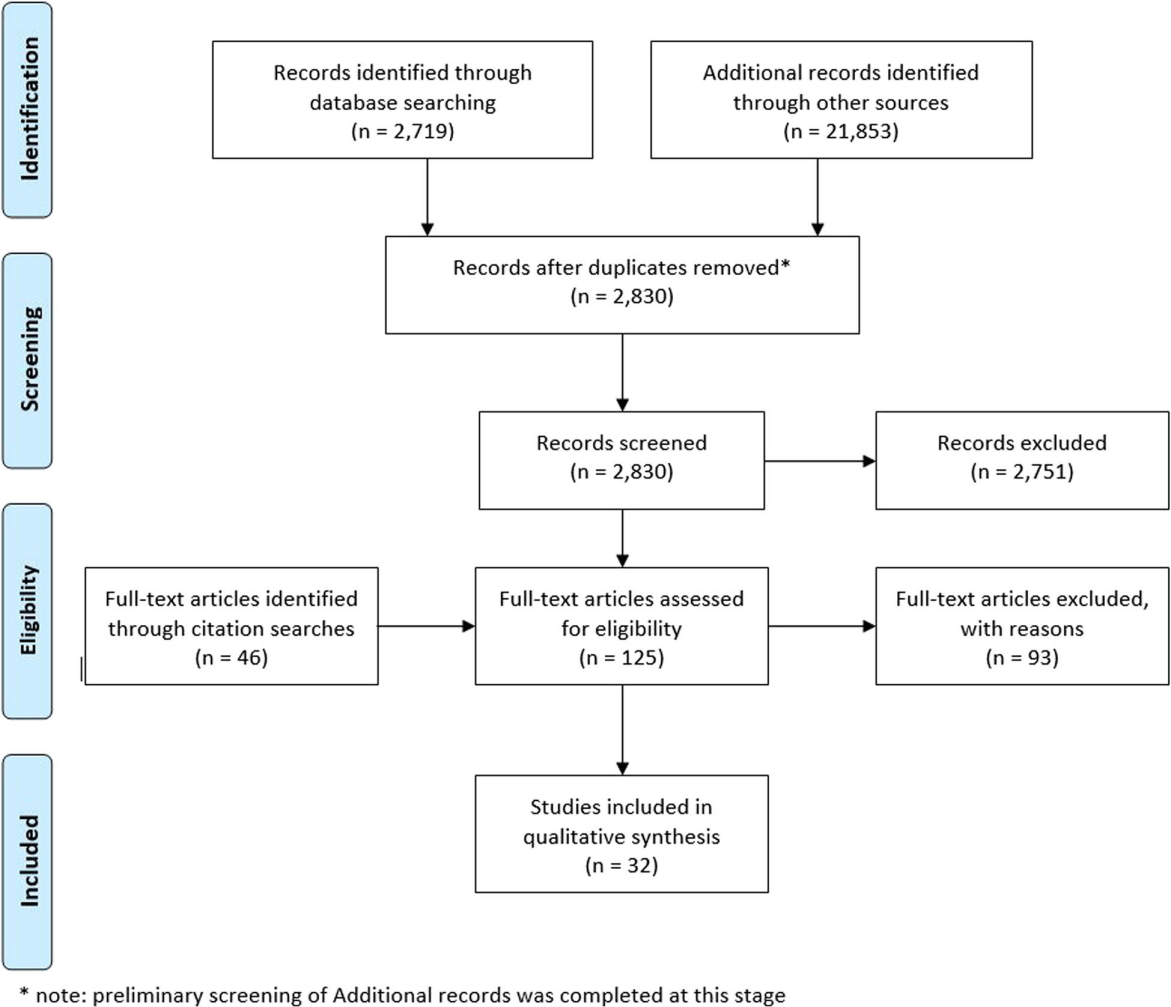
PRISMA flow diagram. PRISMA flow diagram of systematic review literature selection

Database searching yielded 2719 results. After removing duplicates 1258 abstracts were screened and 64 texts were identified for full review. Grey literature searches yielded almost 22,000 results, of which 1369 were screened, with 15 included for full text review. Citation searching led to 310 screened texts, and 46 full text reviews. Thirty-two texts were included for final review (see Fig. 1).

### Literature characteristics and quality assessment

Texts analyzed were primarily journal articles (*n* = 24). Two organizational web articles, a book chapter, a conference paper, a magazine editorial, a United Nations (UN) meeting report, a World Health Organization (WHO) report, and an organizational policy statement were included (see Table 1). Literature was primarily descriptive and conceptual. Based on traditional measures of quality, most of the included literature would be appraised as low quality, as would the evidence base overall.

There was a lack of systematic analysis and original data in the included literature, with most including, at best, narrative reviews of relevant literature. Whilst a number of analyses used illustrative examples to describe CDoH (e.g., Smith, Buse, Gordon [36]) only two presented structured case studies [6, 28].

Eight texts framed analysis through theory and/or frameworks. Two included theoretical descriptions of power. Hastings [9] used de la Boétie’s work on ‘Voluntary Servitude’, exploring power relations relevant to public health in modern society. Madureira Lima and Galea [33] used Lukes’s three-dimensional view of power to study commercial practices that foster consumption, presenting an original framework to study corporate and commercial causes of disease and injury.

Kickbusch et al. [19] presented a specific CDoH framework. Kadandale et al. [28] used this to frame their analysis of the palm oil industry. Sula-Raxhimi et al. [38], drawing on this and an ecological determinants framework, presented a framework for countering corporate power and CDoH, referencing planetary health. Knai et al. [31] described how Meadows’s systems thinking framework may be used to understand CDoH. Freudenberg and Galea [25] included a conceptual model of the influences of corporate practices on health. Buse et al. [16] used an existing framework to classify commercial sector involvement in global governance for health.

### Defining commercial determinants

No widely accepted CDoH definition was apparent. Most (*n* = 19) texts provided no definition. Three prominent definitions were identified.

Most simply, CDoH were termed “factors that influence health which stem from the profit motive” by West and Marteau [42]. This definition was referenced in three other texts [19, 27, 30]. Kickbusch et al. [19] emphasized that this definition fails to distinguish between companies selling health-harming and health-promoting products. Kickbusch et al. [19] instead defined CDoH as “strategies and approaches used by the private sector to promote products and choices that are detrimental to health”, emphasizing that this definition conceptually ties together both macro- (i.e., globalization, global risk society, and global consumer society) and micro- (i.e., individualization, choice and consumer health behavior) concepts, emphasizing these as ‘dynamics’. This definition was used in six other texts [23, 24, 26, 28, 40, 44].

Kosinska and Ostlin [32] provided a broader ‘working definition’ of CDoH that considered “a good or a service where there is an inherent tension between the commercial and the public health objective”, including where the public health imperative is to reduce use or consumption and the commercial imperative is to increase this, or, conversely, where the public health objective is to increase accessibility and affordability and the commercial objective is to reduce this. The first two definitions describe CDoH as broad, systemic factors and dynamics that shape health. However, the third definition frames CDoH as arising from products and services specifically. This definition was also included in a WHO report on governance for children and adolescents’ health and well-being [45].

### Macro-level conditions constituting CDoH

#### Power

Most commonly, CDoH were described as resulting from expressions of economic and political power wielded by large corporate entities, described as “powerful economic operators” [16, 19, 30, 35]. Power imbalances were described both between corporations (large, for-profit, often trans-national entities) and governments with conflicting interests [30, 33, 34, 39, 43], and between corporations and individual citizens, driving behaviors that harm health [6, 33, 39].

Corporate power was said to influence decision making, with corporations sometimes directly involved in public health policymaking [31]. Buse and Hawkes [13] described power as being used to maintain the status quo and deliberately keep ‘difficult’ topics off the agenda. Kickbusch [18] discussed transnational companies’ power to influence political decision-making as largely underestimated. Meanwhile, others argued that CDoH are founded upon unchecked and unseen power exerted by corporations who frame dominant health narratives and agendas [23, 25, 34, 38]. This form of power was described as one of three proposed dimensions of power, alongside power to set agendas and make decisions, and power over conflict [33].

Overall, it was emphasized that powerful private sector interests commonly prevail over public health governance and accountability measures [5, 16, 38, 39]. The source of this power was reportedly changing patterns of global business and consumption, led by rising demand, increasing market coverage, and internationalization of trade and investment [18, 19].

#### Other macro-level conditions constituting CDoH

Social constructs including ideology, neoliberalism and capitalism, globalization, trade agreements, corporate structures and rights, and regulation, were discussed as other macro-level conditions of CDoH.

Kickbusch [18] asserts that

“It has become common practice to turn a health challenge into a fundamental debate about individual freedom and choice. Because health is at the intersection of values and ideology, between market forces and ‘the state’.”

Corporations reportedly favor personal responsibility for health over regulation [6, 27, 33, 34]. Hastings [5] suggested public health professionals need to drive a swing away from corporate capitalism towards economic systems that better promote public health. Others emphasized the role of neoliberal systems overall, and the importance of addressing these in the interests of improved health [9, 39].

CDoH were described as products of contemporary macroeconomics, facilitated by globalization and trans-nationalization of corporations. Many described the globalization of trade and investment, including increased activity within low and middle-income countries, as driving harm and challenging efforts to address CDoH effectively [16, 18, 19, 23, 24, 28, 29, 31, 40, 43, 44]. Notably, food and tobacco industries were cited as the “most internationalised businesses in the entire economy” [24].

Trade agreements and liberalization were described as contributing to worsened health outcomes [16, 24, 31, 34, 36, 41, 43, 44]. The consequences of such agreements relate to occupational conditions, environmental conditions, health systems coverage, tax revenue lost via deregulated global finance systems, the affordability of pharmaceuticals, and national food systems and diets [24, 25, 33, 34, 41]. However, others emphasized that corporations could be more effectively used in prevention efforts, such as through corporate social responsibility (CSR) programs [15, 23, 43].

Overall, corporate structures and rights were critiqued as being, predominantly, at odds with public health due to profit maximization imperatives [5, 9, 23–25, 31, 38, 39]. West and Marteau [42] argue “The greatest challenge to improving health may lie in the tension between wealth- and health-creation”. Wiist [43] emphasized that health-harming products and services are reflective of corporations’ legal responsibilities to investors.

Corporate rights, including intellectual property rights, were described as presenting challenges to many interventions that could benefit public health [16, 33, 34, 43]. Meanwhile, corporations being afforded similar rights as individuals, but with limited liability, was described as contributing to public health harms, and promoting unethical practice [25, 33, 43].

Regulation of corporations contributing to CDoH was reportedly inadequate for preventing ongoing harm [14–16, 27, 31, 33, 39]. Self-regulation by industry was perceived as being the prominent model of regulation [16, 31, 33]. Corporations reportedly stave off public regulation via self-regulation [25, 33] and other methods [16, 25, 27, 30, 31, 33, 34, 37, 39, 43].

### Groups targeted by corporate activities

Groups described as being targeted by corporate actions within CDoH systems included: individual consumers [6, 9, 25, 33], groups living in vulnerable circumstances, including children [6, 24–27, 31, 33, 45]; public health professionals and organizations [25, 33]; researchers and research organizations [6, 25, 33]; philanthropic organizations [33]; not-for-profit organizations [33]; special interest groups and civil society [33]; the WHO and the UN more broadly [16, 33]; and government representatives [5, 6, 9, 16, 25, 39]. These groups were also, at times, described as promoting the interests of commerce and CDoH indirectly through their core activities. For instance, ‘industry friendly’ opinion leaders active within these organizations, philanthropic and/or sponsorship activities, and others, can shape research and policy agendas [33]. Concerns over managing these and other potential conflicts of interest were raised by several authors [16, 23, 31, 32].

### Social, economic and commercial structures, relations and activities through which CDoH manifest

Core structures, relations, and activities through which CDoH manifest included marketing, corporate political activities (CPA) (such as lobbying, litigation, political donations, political relationship building, etc.), CSR, extensive and highly integrated supply chains, products and production detrimental to health, and the accessibility of such products.

Marketing and advertising of unhealthy commodities were widely described as harming health [6, 9, 15, 24, 31, 37] and enhancing the desirability and acceptability of unhealthy commodities [19, 23, 26–28, 35, 40]. Concerns over marketing to children were particularly prominent [6, 15, 24, 28, 31, 45]. One article described corporate marketing as a pathogen [9]. Freudenberg and Galea [25] emphasized that corporations drive consumption by misrepresenting their products’ health consequences and targeting vulnerable populations.

Corporations reportedly continue to spend significantly on marketing [6, 16], allowing unlimited access to consumers [44]. Some described marketing as being used to ‘disguise corporations as friends’ and to position industry as ‘part of the solution’ [5, 37]. Ireland et al. [27] described the “visibility and widespread appeal of sports” as frequently used to promote brands and products that harm health. Through media marketing agreements, corporations were said to gain influence over issues covered on media networks, and therefore over broader health and social narratives [33, 34].

Comprehensive regulation was described as the only strategy likely to effectively reduce the effects associated with marketing [9, 27, 40].

CPA was described variously. Lobbying was regarded as a prominent barrier to healthy public policy, and often used to oppose policies beneficial to public health at the expense of corporate profits [5, 6, 9, 15, 18, 19, 23, 25, 26, 28, 33–35, 38, 39, 42]. Lobbying directly from industry, and indirectly via other groups including think tanks and front groups was also discussed [33, 37].

Litigation, or threatening litigation, was another tactic described as being used against governments seeking to implement policies that might reduce industry profits [6, 16, 25, 33, 42].

Arguments about infringements of personal choice and freedom of speech [15, 34, 40] and obfuscation of scientific evidence through research community capture [6, 25, 33, 34] were cited as obstructing policy processes.

Other CPAs described included lucrative ‘revolving door’ arrangements shuffling individuals between government and commercial sectors, and political donations. Further, participation in government agencies, commissions, committees, and partnerships, pressures on international trade arrangements, and illegal activities were also discussed [5, 6, 16, 25, 27, 33, 34, 38, 39].

CSR is reportedly used to deflect attention from questionable practices, ‘whitewash tarnished reputations’ [19, 26, 35], and enhance credibility and public perception [5, 9, 16, 19, 23, 27, 28, 33, 37, 40]. These include voluntary activities that can undermine or delay official activities [33]. Millar [15] emphasized that while ‘bad’ corporations merely use CSR to offset the damage they do or raise their own profiles, ‘good’ corporations genuinely embrace CSR. Collins et al. [23] described public-private partnerships as opportunities for ‘win-win’ CSR scenarios through shared value creation. However, Kadandale et al. [28] highlighted that partner agencies risk becoming complicit in harmful practices.

Product formulation and production processes reportedly have significant impacts on health [6, 9, 15, 16, 25, 31, 36]. This was articulated as being attributable to corporations’ increased investment in less healthy but more profitable products, added features that increase profits but harm health, resistance to inclusion of features that enhance health but add production costs, population targeting, and lax safety testing [9, 15, 25]. Others allege that product reformulation has often been used as a regulation avoidance tactic [33, 37]. Production processes also reportedly contribute significantly to diminished worker health and wellbeing [25, 28, 34].

The extensive and highly integrated supply chains of modern companies were seen as amplifying influence globally [9, 19, 23, 24, 26, 28, 31, 35, 40, 43]. This has reportedly affected consumption due to the abundance of unhealthy products, relative scarcity of healthy products, and low prices and high profit margins of unhealthy products compared to high costs and lower profit margins of healthy products [24, 25, 34]. This has also led to targeting vulnerable populations [5, 15, 25, 33].

To achieve elevated profits, corporations may externalize costs (environmental, health-related and otherwise) to avoid capturing the true aggregate ‘cost’ of their products [5, 6, 9, 15, 25, 33, 34, 43].

### Consequences of CDoH

Downstream consequences of CDoH were consistently described as premature death and disability associated with NCDs and chronic diseases including ‘industrial epidemics’ and ‘profit driven’ epidemics [5, 6, 9, 13–16, 24, 29–31, 33, 37, 40]. Cancers including lung cancer, obesity and overweight, cardiovascular diseases, chronic obstructive pulmonary disease, high cholesterol, diabetes, cirrhosis, and others were highlighted, as well as injuries. Buse and Hawkes [13] described vaccination and other pharmaceutical development and pricing as contributing to communicable disease outcomes.

Harms to population health were described as outcomes of ‘toxic’ environments. Kickbusch et al. [19] argued that “Health outcomes are determined by the influence of corporate activities on the social environment in which people live and work” emphasizing that environments shape individual lifestyles and choices that determine health outcomes. The International Federation of Medical Students’ Associations reiterated this [26] whilst Franz and Kickbusch [24] stressed “the argument that consumers can decide for themselves does not resonate” given the global consumer society context.

These environments were said to lead to malnutrition, stunting, overweight, obesity, and diabetes within the same populations [44]. Emerging global economies [14, 16, 23, 26], and the poor and “fragile middle” [14] countries were said to be worst affected.

Consequences for physical environments and planetary health associated production and trade included land clearing, lost biodiversity, air pollution, respiratory and cardiovascular diseases, and labor practices including child labor and inadequate maternity protections [28], and pollution, climate change and planetary health [38]. These articles also raised concerns for CDoH consequences for women in particular [28, 38].

### Proposals for harm minimization, and hierarchy of harmful industries

CDoH literature most often referenced the food industry. However, the tobacco and alcohol industries were also frequently described. Pharmaceutical, automotive, firearms, mining and gambling industries were discussed to a lesser extent (see Table 2).

**Table 2:** Included industries. Industries described in the literature reviewed

Author (Year)	Food	Alcohol	Gambling	Tobacco	Pharmaceutical	Automotive	Firearm	Mining	Other
Buse & Hawkes (2015) [13]	x	x		x					
Buse, Tanaka & Hawkes (2017) [16]	x	x		x					
Collins, Mikkelsen, & Axelrod (2019) [23]	x	x		x	x		x		x
Franz & Kickbusch (2018) [24]	x	x		x					
Freudenberg & Galea (2008) [6]	x	x		x	x	x	x		
Freudenberg & Galea (2007) [25]	x	x		x	x	x	x		
Hastings (2015) [9]	x	x		x	x				
Hastings (2012) [5]	x	x		x	x				
International Federation of Medical Students’ Associations, Team of Officials (2017) [26]	x	x		x					
Ireland et al. (2019) [27]	x	x	x	x		x			x
Kadandale, Marten, & Smith (2019) [28]	x	x		x					
Kickbusch (2015) [14]	x	x		x					
Kickbusch (2013) [29]	x	x		x					
Kickbusch (2012) [18]	x	x		x				x	x
Kickbusch, Allen & Franz (2016) [19]	x	x		x					
Kickbusch & Szabo (2014) [30]	x	x		x					
Knai et al. (2018) [31]	x	x	x	x					
Kosinska & Ostlin (2016) [32]	x	x		x	x				
Madureira Lima & Galea (2018) [33]	x	x		x	x	x			
McKee & Stuckler (2018) [34]	x	x		x	x	x	x		
Millar (2013) [15]	x	x	x	x	x		x	x	x
Public Health Association of Australia (2018) [35]	x	x		x					
Smith, Buse & Gordon (2016) [36]	x			x	x				
Smith, Dorfman, Freudenberg, Hawkins, Hilton, Razum & Weishaar (2016) [37]	x	x		x					
Sula-Raxhimi, Butzbach, & Brousselle, (2019) [38]				x					
Thorn (2018) [39]	x	x	x	x					
Thurley (2017) [40]	x	x		x					
United Nations Department of Economic and Social Affairs, and United Nations Industrial Development Organization (2016) [41]	x								
West & Marteau (2013) [42]	x	x	x	x		x		x	x
Wiist (2006) [43]	x	x		x	x	x	x		
World Health Organization (2017) [44]	x								
World Health Organization Regional Office for Europe (2016) [45]	x	x		x	x				x

“x” denotes where the industry has been mentioned at least once within the text

“other” industries included health technology, sporting goods and fitness, built environments, media and information technologies, healthcare [24], sport [26], textiles, energy, water [41], pornography, forestry, gaming, illicit drugs, helmet usage and others [15], oil [30], and health services [35]

Source: Texts included for systematic review

Tobacco industry discussions often referenced the WHO Framework Convention on Tobacco Control (FCTC), comparing it with other industries. Kickbusch [14] emphasized that while FCTC implementation has commenced, few governments have begun counteracting the influence of other unhealthy commodity industries.

Authors described a ‘hierarchy’ of harmful industries, where tobacco is portrayed as the ‘worst’ industry, whilst others were ‘not as bad’. Kickbusch [18] and Ireland et al. [27] included examples from Fédération Internationale de Football Association (FIFA) who vetoed tobacco, yet regard alcohol as integral to the FIFA World Cup. This hierarchy was described as advantaging some industries in promoting their products, and discouraging government intervention. This was said of alcohol, food and gambling industries, compared to tobacco [42].

Some argued that practitioners should view tobacco, alcohol and processed food industries as equivalents, noting unfavorable outcomes associated with alcohol and obesity as “often in a magnitude comparable to that of tobacco” and health-related costs as similar and “perhaps highest for obesity, rather than for tobacco” [37].

Others highlighted the distinction between industries within WHO. Buse et al. [16] assert that

“WHO’s institutional commitment to preventing and managing conflicts of interest with industry is unambiguous, but the scope of the challenge in relation to commercial determinants of NCDs may be impossible to govern”.

Thus, WHO’s financial insecurity may be seen as possibly encouraging some forms of industry engagement, despite their stance on tobacco.

Authors appraised existing efforts to address CDoH as inadequate. Buse et al. [16] noted “While piecemeal efforts have been established, we argue that mechanisms to control the commercial determinants of NCDs are inadequate and efforts at remedial action too limited.” Overall, the need for a new approach and/or paradigm shift to address CDoH harms was emphasized [5, 13, 16, 24, 29, 38, 42].

The need for collaboration beyond single health issues [6, 18, 31] and across sectors was discussed [16, 28, 30, 32, 36, 44, 45]. McKee and Stuckler [34] described the need to “address the power imbalance between global corporations, which are accountable only to their owners and shareholders, and governments, which are accountable to their citizens.” Others reiterated these sentiments [24, 31, 38, 39]. Wiist [39] took this further, suggesting the need to restructure corporate entities, repeal corporate charters, remove corporate personhood rights, and restore liability to shareholders and officials. Sula-Raxhimi et al. [38] suggested a need to find solutions outside the corporate wealth logic mechanisms.

Whilst much of the rights discussion focused on corporate legal and commercial rights, including trade, intellectual property, freedom of speech, and limited liability rights, some supported a reorientation towards human rights and social justice in order to achieve sustainable population health and wellbeing [13, 16, 28, 30, 36, 43].

## Discussion

CDoH are described as underpinning many global health challenges. The CDoH literature ties together macro-level conditions such as economic and political systems, globalization, trade, power dynamics, corporate structures including rights and responsibilities, and regulatory and accountability approaches, with lower-level activities, structures and relations of corporations and related industry groups. However, much of this literature lacks specificity.

No CDoH definition has been consistently applied in the literature. Many authors fail to provide any definition, seemingly assuming some implicit understanding of CDoH. The apparent discord between three definitions identified, whereby West and Marteau [42] emphasize health outcomes arising from the ‘profit motive’, Kickbusch et al. [19] emphasize the promotion of products and choices detrimental to health, and Kosinska and Ostlin [32] describe the tension between commercial and public health objectives specifically for goods and services, highlights a lack of precision within the CDoH literature. This may reflect the dynamic and reflexive nature of the relationships that constitute commercial influences on health.

Further, the CDoH term is not consistently applied. Some texts refer to corporations as elements of SDoH [6, 25] or disease [43], and to commercial interests as distal, structural, societal factors causing disease and injury [33]. Conceptually, these terms and associated discussions closely reflect CDoH, and texts were accordingly included for review. However, other texts were excluded for failing to utilize CDoH language and/or for failing to acknowledge macro-level conditions and/or associated structures, relations and activities as determinants of health and disease.

Many activities, such as marketing [8, 48, 49], CSR [8, 48], and, CPA [7, 49, 50] have been well-documented for their influence on behaviors and health. However, these have largely been studied in isolation, without considering the broader social, economic and political conditions facilitating them, and, at times, without regard for associated outcomes. Similarly, significant literature describes issues such as trade relations [51, 52], globalization [53, 54], health commercialization [55], conflicts between corporate and human rights [55, 56], and health-harming products [53, 57] without acknowledging these as determinants of health or CDoH specifically. This represents a lacuna in the evidence base.

Whilst CDoH outcomes are mostly described as harms, Millar [15] proposed that some corporate entities ‘do real good’ for the sake of doing good, whilst others highlighted the perception that some industries are not as harmful as others. McKee and Stuckler [34] indicated that corporations can be a ‘force for good or bad’, dependent on their activities and partnerships. These interpretations may suggest that CDoH could be positive or negative, with benefits and harms nuanced and circumstantial. We question whether harm and ill-health are defining consequences of CDoH, or whether there could be scenarios, presently or in future, where commercial determinants could be consistent with public health interests and positively influence health. That is whether, as with SDoH, CDoH occur along a gradient or nuanced spectrum, thereby influencing population health and wellbeing negatively and/or positively depending on the context. Whilst current literature focuses on negative outcomes it is possible that positive CDoH outcomes may eventuate with appropriate intervention and/or context. This warrants further exploration. In particular, this work could consider the CDoH from a systems perspective, recognize the influence of CDoH at various levels, and explore the structures and, most importantly, the reflexive relations that generate environments, conditions and behaviors that shape health and wellbeing. A new CDoH definition that considers these factors may assist in addressing the current lack of precision in the literature base, whilst also going some way to framing responsive and reflexive CDoH interventions in future.

This review documents the macro-level conditions, relations, structures and activities, and consequences constituting CDoH as described in the CDoH literature. That is, literature self-identified as describing CDoH and associated concepts. Given the nascent nature of the CDoH literature, this review provides a timely synthesis of the current state of understanding.

Some limitations apply to this review. The narrow searching frame may have meant that some literature pertaining to peripheral CDoH concepts may not have been captured in this review. Further, the working definition adopted for initial planning may present a potential limitation [19].

Reviewed literature was limited to that published in English only. As such, perspectives from high-income countries frame much of the literature. However, material reviewed also discusses implications for low- and middle-income countries, including understanding and concern for the flow of commercial influences into these countries [5, 14, 16, 19, 23, 26, 28, 34, 38].

There are some inherent limitations to grey literature searching given the volume of results and the ‘filter bubble’, generated by search engines that tailor results to individual search histories and preferences. However, the inclusion of grey literature allowed for a more comprehensive review.

CDoH present an emerging, yet relatively underdeveloped, area of academic interest and concern. There is limited capacity to synthesize substantive findings, as these are not yet developed in the CDoH-specific literature. So far, the literature has largely focused on describing, rather than addressing, harm. As such, approaches to preventing the harms associated with CDoH are largely hypothetical, with some important exclusions including tobacco control efforts. It is anticipated that the literature base will continue to expand over coming years, with future work beginning to explore this further in the context of CDoH specifically.

## Conclusions

The CDoH field is of increasing social and public health relevance. Whilst the literature base is in its infancy, it has begun to illustrate the multiple elements constituting CDoH, groups most affected, and resulting diminished population health outcomes. Overall, there is a need for greater specificity in the CDoH literature. As there is no widely accepted CDoH definition in use, evolution of this may be pertinent. Further, CDoH language should be more widely adopted to describe corporate influences on health and mechanisms reinforcing these globally, to better recognize these as significant contributors to global disease. In order to reduce NCDs and improve population wellbeing globally, systematic approaches to identifying, describing, and where necessary disrupting the complex conditions constituting CDoH are needed.

## Supplementary information

**Additional file 1.** Search strategies. Complete commercial determinants of health search strategies from database and grey literature searches.

## Data Availability

Not applicable.

## Abbreviations

CDoH: Commercial determinants of health
CPA: Corporate political activity
CSR: Corporate social responsibility
FCTC: Framework Convention on Tobacco Control
FIFA: Fédération Internationale de Football Association
NCD: Non-communicable disease(s)
SDoH: Social determinants of health
UN: United Nations
WHO: World Health Organization
