# Supplementary material for: Defining the commercial determinants of health: a systematic review
Source: BMC Public Health. 2020 Jun 29;20:1022. doi: 10.1186/s12889-020-09126-1 (PMC7325018; doi:10.1186/s12889-020-09126-1)
Supplement: Supplementary file 1 — Additional file 1. Search strategies. Complete commercial determinants of health search strategies from database and grey literature searches. [file 12889_2020_9126_MOESM1_ESM.docx]

**Additional file 1 - Search strategies**

**Complete commercial determinants of health search strategies from database and grey literature searches**

|  | **Platform or URL** | **Date** | **Search type and/or limitations** | **Search terms** | **Results** |
| --- | --- | --- | --- | --- | --- |
| **Database searching** | | | | | |
| Medline | Ovid | 15 May 2018 | Keyword (.mp limitation) | (commercial OR corporate) AND determinant* AND (health OR disease*) | 388 |
| Embase | Ovid | 15 May 2018 | Keyword (.mp limitation) | (commercial OR corporate) AND determinant* AND (health OR disease*) | 798 |
| Scopus | N/A | 15 May 2018 | Keyword (.mp limitation) | (commercial OR corporate) AND determinant* AND (health OR disease*) | 895 |
| Global Health | N/A | 15 May 2018 | Keyword (.mp limitation) | (commercial OR corporate) AND determinant* AND (health OR disease*) | 638 |
| **Grey literature searching: Customised Google searches** | | | | | |
| Google Advanced Search | https://www.google.com/advanced_search | 13 June 2018 | Google Advanced Search | “Commercial determinants of health” | 3,540 |
| Google Advanced Search | https://www.google.com/advanced_search | 13 June 2018 | Google Advanced Search | “Commercial determinant of health” | 26 |
| Google Advanced Search | https://www.google.com/advanced_search | 13 June 2018 | Google Advanced Search | “Commercial determinants of disease” | 5 |
| Google Advanced Search | https://www.google.com/advanced_search | 13 June 2018 | Google Advanced Search | “Commercial determinant of disease” | 1 |
| Google Advanced Search | https://www.google.com/advanced_search | 13 June 2018 | Google Advanced Search | “Corporate determinants of health” | 41 |
| Google Advanced Search | https://www.google.com/advanced_search | 13 June 2018 | Google Advanced Search | “Corporate determinant of health” | 0 |
| Google Advanced Search | https://www.google.com/advanced_search | 13 June 2018 | Google Advanced Search | “Corporate determinants of disease” | 0 |
| Google Advanced Search | https://www.google.com/advanced_search | 13 June 2018 | Google Advanced Search | “Corporate determinant of disease” | 1 |
| **Grey literature searching: Grey literature databases** | | | | | |
| The Community Guide (CDC) | https://www.thecommunityguide.org/ | 13 June 2018 | N/A | (commercial OR corporate) AND determinant AND (health OR disease) | 57 |
| National Institute for Health and Care Excellence (NICE, UK reviews) | https://www.nice.org.uk/ | 13 June 2018 | N/A | (commercial OR corporate) AND determinant* AND (health OR disease*) | 67 |
| Centre for Reviews Dissemination (University of York) | https://www.york.ac.uk/crd/ | 13 June 2018 | N/A | (commercial OR corporate) AND determinant* AND (health OR disease*) | 33 |
| Health Evidence | https://www.healthevidence.org/ | 13 June 2018 | N/A | (commercial OR corporate) AND determinant* AND (health OR disease*) | 0 |
| **Grey literature searching: Targeted website searches** | | | | | |
| Asia-Europe Foundation (ASEF) | http://www.asef.org/ | 19 June 2018 | Match all keywords limitation | Commercial determinant health | 0 |
| Asia-Europe Foundation (ASEF) | http://www.asef.org/ | 19 June 2018 | Match all keywords limitation | Commercial determinant disease | 0 |
| Asia-Europe Foundation (ASEF) | http://www.asef.org/ | 19 June 2018 | Match all keywords limitation | Corporate determinant health | 2 |
| Asia-Europe Foundation (ASEF) | http://www.asef.org/ | 19 June 2018 | Match all keywords limitation | Corporate determinant disease | 0 |
| Asia-Pacific Academic Consortium for Public Health | http://www.apacph.org/ | 19 June 2018 | N/A | (commercial OR corporate) AND determinant* AND (health OR disease*) | 0 |
| Association of Schools of Public Health in the European Region (ASPHER) | https://www.aspher.org/ | 19 June 2018 | N/A | (commercial OR corporate) AND determinant* AND (health OR disease*) | 0 |
| Centers for Disease Control and Prevention (CDC) | https://www.cdc.gov/ | 19 June 2018 | N/A | (commercial OR corporate) AND determinant* AND (health OR disease*) | 6 |
| EuroHealthNet | https://eurohealthnet.eu/ | 19 June 2018 | N/A | (commercial OR corporate) AND determinant* AND (health OR disease*) | 719^#^ |
| European Centre for Disease Prevention and Control (ECDC) | https://ecdc.europa.eu/en/home | 19 June 2018 | N/A | (commercial OR corporate) AND determinant* AND (health OR disease*) | 7,855^#^ |
| European Commission | https://ec.europa.eu/commission/index_en | 19 June 2018 | N/A | (commercial OR corporate) AND determinant* AND (health OR disease*) | 23 |
| European Observatory on Health Systems and Policies | http://www.euro.who.int/en/about-us/partners/observatory | 19 June 2018 | N/A | (commercial OR corporate) AND determinant* AND (health OR disease*) | 1 |
| European Public Health Alliance (EPHA) | https://epha.org/ | 19 June 2018 | N/A | Commercial determinant health | 22 |
| European Public Health Alliance (EPHA) | https://epha.org/ | 19 June 2018 | N/A | Commercial determinant disease | 15 |
| European Public Health Alliance (EPHA) | https://epha.org/ | 19 June 2018 | N/A | Corporate determinant health | 8 |
| European Public Health Alliance (EPHA) | https://epha.org/ | 19 June 2018 | N/A | Corporate determinant disease | 3 |
| The Graduate Institute Geneva | http://graduateinstitute.ch/home.html | 19 June 2018 | N/A | (commercial OR corporate) AND determinant* AND (health OR disease*) | 1 |
| Institute for Health Metrics and Evaluation (IHME) | http://www.healthdata.org/ | 19 June 2018 | N/A | (commercial OR corporate) AND determinant* AND (health OR disease*) | 2,272^#^ |
| IOGT International | http://iogt.org/ | 19 June 2018 | N/A | Commercial determinant health | 14 |
| IOGT International | http://iogt.org/ | 19 June 2018 | N/A | Commercial determinant disease | 12 |
| IOGT International | http://iogt.org/ | 19 June 2018 | N/A | Corporate determinant health | 11 |
| IOGT International | http://iogt.org/ | 19 June 2018 | N/A | Corporate determinant disease | 10 |
| NCD Alliance | https://ncdalliance.org/ | 19 June 2018 | N/A | (commercial OR corporate) AND determinant* AND (health OR disease*) | 3 |
| Public Health Association of Australia (PHAA) | https://www.phaa.net.au/ | 19 June 2018 | N/A | Commercial determinant health | 2 |
| Public Health Association of Australia (PHAA) | https://www.phaa.net.au/ | 19 June 2018 | N/A | Commercial determinant disease | 2 |
| Public Health Association of Australia (PHAA) | https://www.phaa.net.au/ | 19 June 2018 | N/A | Corporate determinant health | 2 |
| Public Health Association of Australia (PHAA) | https://www.phaa.net.au/ | 19 June 2018 | N/A | Corporate determinant disease | 1 |
| Public Health Association of BC | https://phabc.org/ | 19 June 2018 | N/A | (commercial OR corporate) AND determinant* AND (health OR disease*) | 498^#^ |
| VicHealth | https://www.vichealth.vic.gov.au/ | 19 June 2018 | N/A | (commercial OR corporate) AND determinant* AND (health OR disease*) | 1,590^#^ |
| World Health Organization (WHO) | http://www.who.int/ | 19 June 2018 | N/A | Commercial determinant health | 1,030^#^ |
| World Health Organization (WHO) | http://www.who.int/ | 19 June 2018 | N/A | Commercial determinant disease | 1,030^#^ |
| World Health Organization (WHO) | http://www.who.int/ | 19 June 2018 | N/A | Corporate determinant health | 1,480^#^ |
| World Health Organization (WHO) | http://www.who.int/ | 19 June 2018 | N/A | Corporate determinant disease | 1,470^#^ |
| *Note:*  ** denotes truncation*  *^#^ denotes where first 100 search results have been screened only*  *N/A denotes ‘not applicable’* | | | | | |
